# Impact of the subcutaneous formulations of trastuzumab and rituximab on efficiency and resource optimization in Spanish hospitals: H-Excelencia study

**DOI:** 10.1186/s12913-021-06277-8

**Published:** 2021-04-08

**Authors:** María Reyes Abad-Sazatornil, Ainhoa Arenaza, Juan Bayo, Jesus García Mata, José María Guinea De Castro, Josefa León, Javier Letellez, Virginia Reguero, Carmen Martínez Chamorro, Antonio Salar

**Affiliations:** 1 Hospital Pharmacy, Hospital Universitario Miguel Servet, P/ Isabel la Católica 1-3, 50009 Zaragoza, Spain; 2 Hospital Pharmacy, Hospital Clínico San Carlos, Madrid, Spain; 3 Oncology Unit, Hospital Juan Ramón Jiménez, Huelva, Spain; 4 Medical Oncology Unit, Complexo Hospitalario Universitario de Ourense, Ourense, Spain; 5 Haematology and Haemotherapy Unit, Hospital Universitario de Araba, Vitoria-Gasteiz, Álava Spain; 6 Hospital Pharmacy, Hospital Morales Meseguer, Murcia, Spain; 7 Hospital Pharmacy, Hospital de Fuenlabrada, Madrid, Spain; 8 Hospital De Cabueñes, Gijón, Asturias Spain; 9 Haematology Unit, Hospital Universitario Quirónsalud, Madrid, Spain; 10 Haematology Unit, Hospital de Mar, Barcelona, Spain

**Keywords:** Efficiency indicators, Healthcare capacity, Healthcare quality, Intravenous, Resource optimization, Subcutaneous

## Abstract

**Background:**

Subcutaneous (SC) versus intravenous (IV) administration is advantageous in terms of patient convenience and hospital efficiency. This study aimed to compare the effect of optimizing the processes involved in SC versus IV administration of rituximab and trastuzumab on hospital capacity and service quality.

**Methods:**

This cross-sectional resource utilization study interviewed oncologists, hematologists, nurses, and pharmacists from 10 hospitals in Spain to estimate changes in processes associated with conversion from IV to SC rituximab and trastuzumab, based on clinical experience and healthcare use from administrative databases.

**Results:**

Efficient use of SC formulations increased the monthly capacity for parenteral administration by 3.35% (potentially increasable by 5.75% with maximum possible conversion according to the product label). The weekly capacity for hospital pharmacy treatment preparation increased by 7.13% due to conversion to SC formulation and by 9.33% due to transferring SC preparation to the cancer treatment unit (potentially increasable by 12.16 and 14.10%, respectively). Monthly hospital time decreased by 33% with trastuzumab and 47% with rituximab. In a hypothetical hospital, in which all processes for efficient use of SC rituximab and/or trastuzumab were implemented and all eligible patients received SC formulations, the estimated monthly capacity for preparation and administration increased by 23.1% and estimated hospital times were reduced by 60–66%.

**Conclusions:**

Conversion of trastuzumab and rituximab to SC administration could improve the efficiency of hospitals and optimize internal resource management processes, potentially increasing care capacity and improving the quality of care by reducing time spent by patients at hospitals.

**Supplementary Information:**

The online version contains supplementary material available at 10.1186/s12913-021-06277-8.

## Background

Rituximab (MabThera®; Roche) and trastuzumab (Herceptin®; Roche) were the first monoclonal antibodies (mAbs) approved for cancer treatment by the European Medicines Agency (EMA) in 1998 and 2000, respectively [1, 2]. The introduction of these drugs revolutionized the treatment of hematological and solid malignancies, with both becoming the standard of care for their respective indications. Both mAbs were first developed as intravenous (IV) formulations with weight-dependent dosing. Subsequently, Roche developed subcutaneous (SC) formulations of rituximab and trastuzumab by concentrating the IV formulations and adding recombinant human hyaluronidase, which enabled SC delivery of effective drug volumes [3]. Preclinical and clinical trials demonstrated that the IV and SC formulations of both drugs had similar pharmacokinetic, efficacy, and safety profiles [4–9]. As a result, the EMA approved SC trastuzumab for early and metastatic breast cancer in 2013, and SC rituximab for non-Hodgkin’s lymphoma in 2014 [10, 11]. The Spanish Agency for Medicines and Health Products approved SC trastuzumab in 2014 and SC rituximab in 2015.

Unlike their IV formulations, SC rituximab and SC trastuzumab offer a shorter drug administration time (< 5 min) and contain fixed doses administered by a single-use injection device; therefore, the dosages are independent of the patient’s body weight [10, 11]. In addition, there is no need for a loading dose with SC trastuzumab [11]. Because of the need for weight-based dosing, IV doses of rituximab and trastuzumab must be prepared in hospital pharmacies using a vertical laminar flow hood in order to ensure the quality of the active substance, which is not necessary with the SC formulations. As a result, both drug preparation and administration times are shorter with the SC than with the IV formulations, which has led to relevant economic benefits [12–17], as well as a positive impact on patients’ drug preferences [18–21] and quality of life [17, 22]. Altogether, these benefits may also improve the efficiency of onco-hematology hospitals by optimizing healthcare resource utilization and reducing the patients’ duration of hospital stay.

The primary objective of the present study was to quantify the effects of SC versus IV formulations of rituximab and trastuzumab on the efficiency of Spanish hospitals in terms of healthcare capacity and quality of the services. As a secondary objective, we estimated the impact of SC versus IV treatment on the efficiency of a theoretical hospital that optimizes all of the processes identified in the first part of the study.

## Methods

This was a two-phase, observational, cross-sectional study, in which participants (hematologists, oncologists, hematology nurses, oncology nurses, and hospital pharmacists) were interviewed to collate resource use information.

Five different questionnaires were developed and validated for use during the face-to-face interviews, and were tailored to each type of healthcare professional (HCP) that were interviewed. The questionnaires were used to gather information from the participant HCPs on resource use based on their recent clinical experience with the management of patients receiving rituximab or trastuzumab. The first questionnaire, for pharmacists, focused on the treatment preparation process; the next two were for oncologists and hematologists (specific for trastuzumab and rituximab, respectively), which focused on the prescription process, disease management including visits and monitoring; and the last two were for nurses (one specific for trastuzumab and the other for rituximab), which focused on the administration process including hospital opening hours, number of daily administrations, number of infusion chairs and average occupancy time. See Additional file 1 for the detailed questionnaires.

**Fig. 1:**
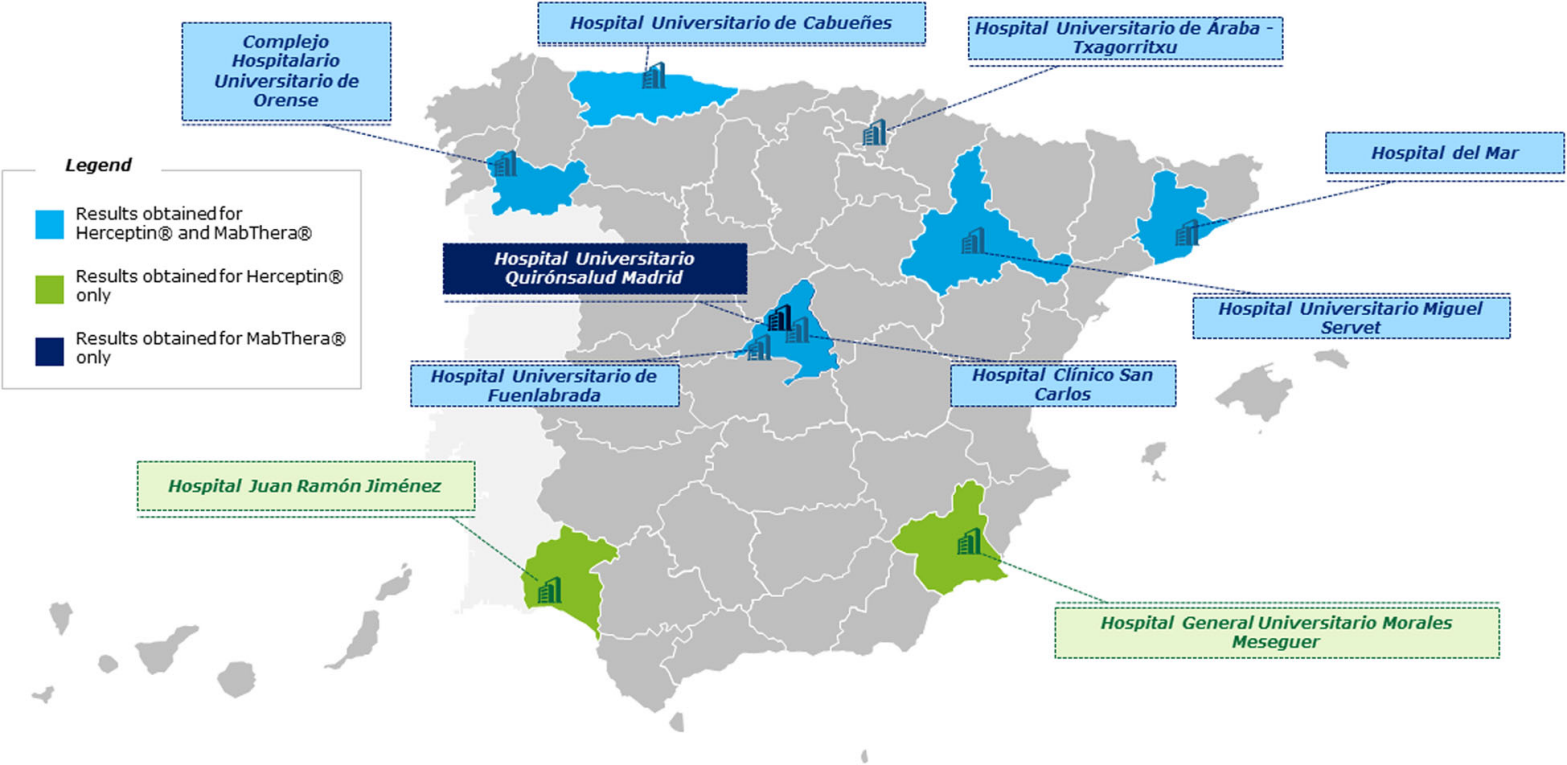
Participating hospitals

In the first phase, fieldwork research was conducted by interviewing HCPs (hematologists, oncologists, nurses, and hospital pharmacists) from reference hospitals in different regions of Spain (Fig. 1). The aim of this fieldwork research was to: (1) identify all of the processes optimized by the use of the SC formulation in these Spanish hospitals; (2) determine performance indicators that would serve as measures to analyze changes in processes, times, volumes, and drug administration due to the SC conversion of both rituximab and trastuzumab; and (3) quantify the improvement in efficiency and healthcare quality as a result of the switch from IV to SC formulations.

From the data collected in the fieldwork, seven parameters were identified that could be used to measure the impact of the identified optimized processes with SC formulations on capacity (four parameters) and quality (three parameters). Capacity parameters were defined as the estimated time spent preparing and administering treatments (Table 1), and quality parameters were defined as the duration of hospital stay, impact on patients’ and/or caregivers’ labor productivity, and the need for a venous access device with a reservoir (Table 1).

**Table 1:** Parameters used to compare the impact of SC vs IV rituximab or trastuzumab administration

Parameter	Method for calculation
**Capacity of the hematology/oncology cancer treatment unit**	
1. Increase treatment administration at the cancer treatment unit due to IV to SC conversion, n (%)	Based on the total time saved with SC and the average time of administration of any drug. ● Total time saved with SC = (Difference in time spent in cancer treatment unit with IV vs SC administration) × (number of patients treated with IV and SC trastuzumab or rituximab) × (no. of visits for IV or SC treatment) ● Average time of administration of any drug [IV and SC trastuzumab, rituximab or other treatment] = based on opening hours, number of positions and volume of daily treatments
2. Increase in treatment administration due to rituximab premedication being administered in waiting room, n (%)	Based on the total time saved in the administration chair and the average time of administration of any drug. ● Total time saved = (Time spent in cancer treatment unit for administration of IV rituximab premedication) × (number of patients treated with rituximab in maintenance) × (no. of visits for rituximab administration) ● Average time of administration of any drug [IV and SC trastuzumab, rituximab or other treatment] = based on opening hours, number of positions and volume of daily treatments
3. Increase in treatments that can be prepared and administered at the reference hospital because patients receiving SC can be treated at hospitals closer to their homes, n (%)	Increase in the number of treatments that can be prepared (based on the total time saved and the average time of preparation of any drug): ● Total time saved with transfer of SC treatment preparation to regional hospital = (Difference in pharmacy preparation time with IV vs SC formulations) × (no. of doses prepared by the regional hospital) ● Average time of preparation of any drug [IV and SC trastuzumab, rituximab or other treatment] = based on opening hours, number of vertical laminar flow hoods and volume of daily treatment preparations Increase in the number of treatments that can be administered (based on the total time saved and the average time of administration of any drug): ● Total time saved by cancer treatment unit with transfer of SC treatment to regional hospital = (Difference in time spent in cancer treatment unit with IV vs SC administration) × (no. of patients referred to regional hospital for treatment) × (no. of visits for IV or SC treatment) ● Average time of administration of any drug [IV and SC trastuzumab, rituximab or other treatment] = based on opening hours, number of positions and volume of daily treatments
4. Increase in treatments that can be prepared in the vertical laminar flow hood at the hospital pharmacy, n (%)	Based on the total time saved due to the reduction in preparation times with SC or since SC formulation can be prepared at the cancer treatment unit and the average time of preparation of any drug. ● Time saved in the hospital pharmacy = (Difference in time spent preparing IV formulation in the vertical laminar flow hood vs preparing SC doses) × (no. of doses prepared) ● Average time of preparation of any drug [IV and SC trastuzumab, rituximab or other treatment] = based on opening hours, number of vertical laminar flow hoods and volume of daily treatment preparations
**Quality**	
1. Reduction in time spent in hospital (SC vs IV), minutes	● Reduction in time spent in hospital = [(Average medical consultation time for IV treatment) – (average medical consultation time for SC treatment)] + [(average wait time to receive IV treatment) – (average wait time to receive SC treatment)] + [(average time spent in the cancer treatment unit with IV administration) – (average time spent in the cancer treatment unit with SC administration)]
2. Improvement in caregiver’s and/or patient’s work productivity, € (%)	● Improvement in labor productivity (assessed by economic- and time-related measures) = (Reduction in time spent in hospital [from row above]) × (average cost of professional per minute [estimated at 0.205464 €/min])^a^
3. Reduction in the use rate and time of venous access devices with reservoirs, n (%)	● (No. of patients requiring a venous access device with a reservoir [port a cath] during IV maintenance treatment) – (no. of patients carrying a venous access device [PICC] during SC maintenance treatment) ● Reduction in time using a venous access device, as PICC requires less time than port-a-cath

^a^Derived from Spanish labor market statistics [23], where the average annual gross salary is €23,022.20, and working hours of 155.6 h per month

*IV* intravenous, *PICC* peripherally inserted central catheter, *SC* subcutaneous

In the second phase of the study, the change in healthcare capacity and quality were estimated for a hypothetical hospital, in which all the identified processes would be implemented and the maximum number of patients would be administered SC formulations of rituximab and trastuzumab, according to their respective approved summary of product characteristics (SmPC). Based on the potential total number of patients eligible to receive either IV rituximab or trastuzumab per indication and selecting only SC-approved indications, it was estimated that 45% of all rituximab doses and 91% of all trastuzumab doses could be administered using the SC formulations.

The impact of SC versus IV treatment on the seven defined parameters were described as number of units (N), percentages (%) or presented in other units of measure (i.e., hours or euros [€]) as required in each case. Aggregated results were calculated using the mean value of the percentage variation of all the hospitals considered for each parameter.

## Results

The study included 10 reference hospitals from across Spain (Fig. 1). Seven hospitals provided data on the use of trastuzumab and rituximab, two provided data only on the use of trastuzumab, and one provided data only on the use of rituximab. At centers administering rituximab (*n* = 8), the proportion of patients who received SC rather than IV infusions of rituximab as part of a chemotherapy regimen ranged from 0.0 to 53.3%, and SC rituximab as part of maintenance therapy ranged from 36.2 to 100.0%. A similar trend was seen with use of SC trastuzumab at the nine centers where this treatment was studied. When used in combination with chemotherapy, between 0.0 and 91.7% of the trastuzumab doses were administered by SC infusion, and when used as part of maintenance therapy, between 39.8 and 100.0% of the trastuzumab doses were given by SC infusion. Overall, hospitals had converted between 31.3 and 96.0% of trastuzumab doses and between 36.6 and 68.2% of rituximab doses from IV to SC administration.

### Identified processes for efficient use of SC administration

During the interviews, five processes were identified that were already implemented by the hospitals to optimize the efficient administration of SC rituximab and SC trastuzumab. These processes were: (1) preferential use of SC over IV treatments, whenever possible, with specific separate protocols in place for SC and IV administration (e.g., a dedicated chair for SC administration within the cancer treatment unit); (2) required oral premedication prior to the use of rituximab administered to the patient while they were in the waiting room; (3) patients receiving SC treatment at their nearest regional hospital (instead of receiving it in the reference hospital), whenever possible; (4) preparation of SC treatments outside the vertical laminar flow hood (at the hospital pharmacy, the cancer treatment unit or the regional hospital pharmacy); and (5) avoidance of venous access devices in patients receiving trastuzumab, whenever possible. Nevertheless, it was assumed that not all of the participating centers were able to implement all of these processes due to their internal therapeutic protocols and established management procedures.

Eight of the 10 hospitals (80.0%) had a dedicated chair within the cancer treatment unit for SC administration only. At the eight centers where rituximab was administered, seven hospitals (87.5%) started the administration of necessary premedication to patients while they were in the waiting room prior to the use of SC rituximab. Two hospitals treated patients from outside their reference areas, of which one of these hospitals (50.0%) had implemented a protocol for preparing and administering SC treatments to patients at their local regional hospital instead of at the reference hospital. Two of the 10 hospitals (20.0%) had established protocols for preparing SC treatments outside the vertical laminar flow hood (at the hospital pharmacy or the cancer treatment unit). Seven of the nine hospitals that administered trastuzumab (77.8%) were able to avoid using venous access devices in patients by converting from IV to SC formulation.

### Impact of SC formulations on hospital capacity

#### Increase in treatment administration at the cancer treatment unit due to IV to SC conversion

Across the 10 hospitals, the conversion of rituximab and trastuzumab treatment from IV to SC administration increased the total number of treatments that were able to be administered monthly by 3.35%, due to reductions in time when the administration chair was occupied.

#### Increase in treatment administration due to rituximab premedication being administered in waiting room

In addition to the increased treatment capacity due to conversion of rituximab and trastuzumab from IV to SC, the total number of treatments that could be administered in the cancer treatment unit increased by 0.31% when premedication of rituximab was administered in the waiting room, which was possible with SC rituximab administration.

#### Increase in treatments that can be prepared and administered at the reference hospital because patients receiving SC can be treated at hospitals closer to their homes

Due to the availability of SC administration, two of 10 centers admitted patients from outside of their reference area, and only one offered the possibility of SC trastuzumab administration at other regional hospitals. The transfer of SC administration for some patients to regional hospitals increased the monthly capacity for treatment administration at the reference hospital by 0.17%. Moreover, at the reference hospital, the weekly capacity of the hospital pharmacy to prepare treatments under the laminar flow hood increased by 0.53% as a result of patient referral to the nearby regional hospitals. Furthermore, patients required notably less travelling time to get the regional hospital.

#### Increase in treatments that can be prepared in the vertical laminar flow hood at the hospital pharmacy

Overall, based on the measured use of SC rituximab and/or trastuzumab at these centers and the total time savings involved in dose preparation, the weekly capacity to prepare treatment under the laminar flow hood increased by 7.13% as a result of the conversion from IV to SC formulation. On the other hand, transferring the preparation of SC formulations to the cancer treatment unit, rather than at the hospital pharmacy, allowed for a 9.33% increase in the weekly capacity to prepare other treatments under the laminar flow hood of the hospital pharmacy.

### Impact on quality of care

#### Reduction in time spent in hospital

**Fig. 2:**
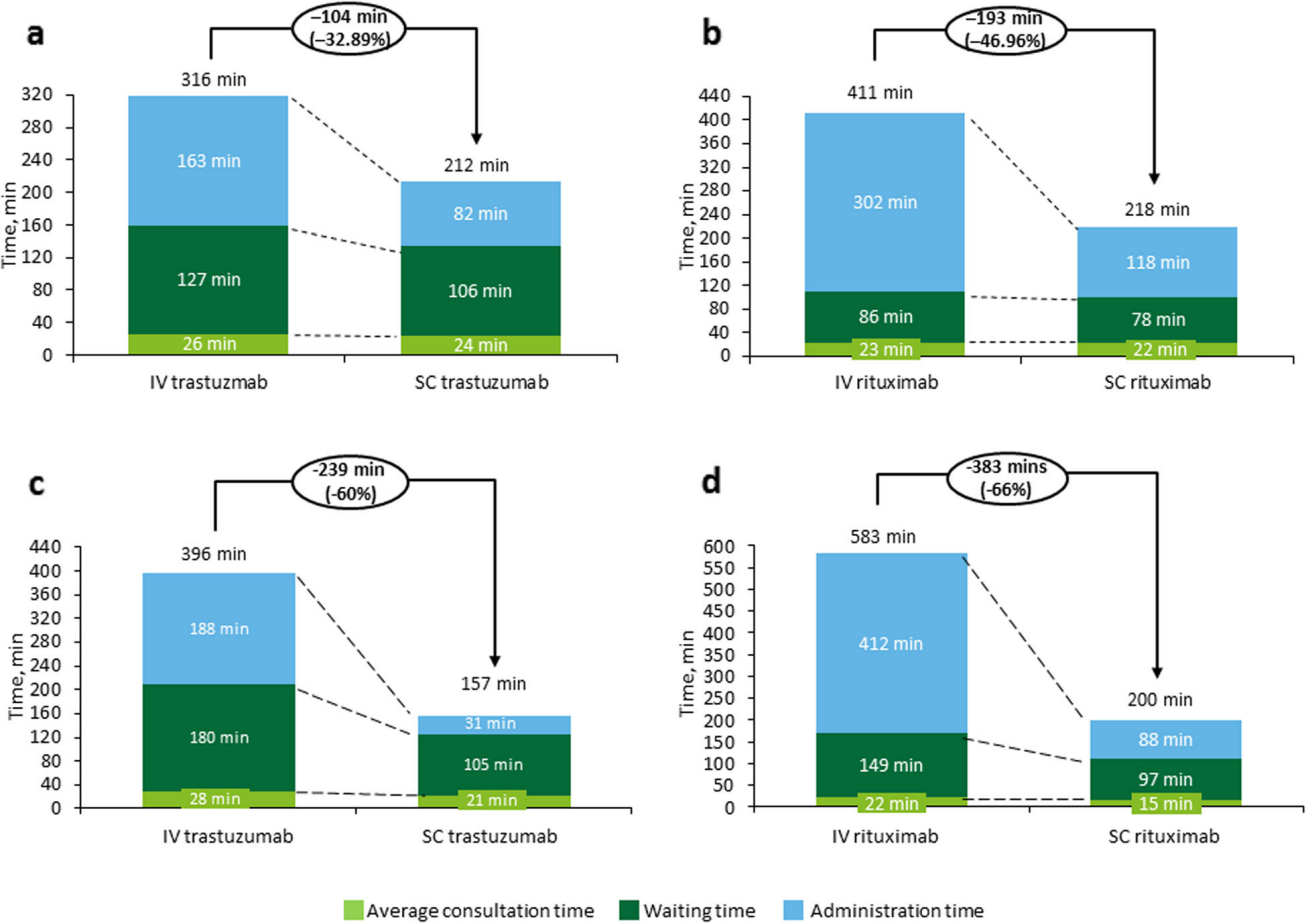
Reduction of time in the hospital as a result of conversion of (**a**) trastuzumab and (**b**) rituximab from IV to SC administration, and as a result of hypothetical maximum conversion of (**c**) trastuzumab and (**d**) rituximab from IV to SC administration. *IV* intravenous, *SC* subcutaneous

The impact of IV to SC conversion on the time spent in hospital differs depending on whether it is administered in combination with chemotherapy or as monotherapy (Table 2). Compared with IV administration, SC administration of trastuzumab was associated with a 32.89% total reduction in the average monthly hospitalization time (Fig. 2a), while SC rituximab was associated with a 46.96% total reduction (Fig. 2b). The most significant change was seen in the time spent by patients in the administration chair; waiting time at the cancer treatment unit and medical consultation times were also reduced, but to a smaller extent.

**Table 2:** Estimated time required per month to administer rituximab or trastuzumab subcutaneously or intravenously

Average time, min	Waiting room		Administration chair		Medical consultation		Total	
	IV	SC	IV	SC	IV	SC	IV	SC
**Rituximab**								
Per cycle in combination with chemotherapy	71	71	296	139	20	20	387	230
Per maintenance treatment cycle	68	53	183	21	16	15	267	89
**Weighted average total time per patient per month**^**a**^	**86**	**78**	**302**	**118**	**23**	**22**	**411**	**218**
**Trastuzumab**								
First cycle (including loading dose) in combination with chemotherapy^b^	83	83	187	83	17	16	286	181
Per cycle in combination with chemotherapy (without loading dose)	85	85	162	107	17	16	264	208
Per maintenance treatment cycle	85	60	64	15	17	16	166	91
**Average total time per patient per month**^**b, c**^	**127**	**106**	**163**	**82**	**26**	**24**	**316**	**212**

^a^The weighted average total time per patient per month was estimated by dividing the total treatment time in a year by 12 months. Total treatment pattern in a year differs per approved indication as follows: (1) First-line follicular lymphoma (48% of patients treated with rituximab): 8 cycles in combination with chemotherapy (the first one always IV) and the last 12 as maintenance therapy; (2) Relapsed refractory follicular lymphoma (14% of patients treated with rituximab): 8 cycles in combination with chemotherapy (the first one always IV) and the last 8 as maintenance therapy; (3) Diffuse large B-cell lymphoma (38% of patients): 8 cycles in combination with chemotherapy (the first one always IV)

^b^Data for the first trastuzumab cycle (i.e. that including the loading dose) were missing from three hospitals, so the average times shown are from six of the nine hospitals

^c^The average total time per patient per month was estimated by first calculating the total treatment time in a year, consisting of a first cycle with loading dose in combination with chemotherapy, 7 subsequent cycles in combination with chemotherapy, and 10 cycles of maintenance therapy (giving a total of 18 cycles/year), and then dividing by 12 months

*IV* intravenous, *SC* subcutaneous

#### Improvement in caregiver’s and/or patient’s work productivity

The reduction in total hospitalization time for patients receiving trastuzumab and rituximab, both in combination with chemotherapy and as maintenance therapy, led to a 21.2% average increase in caregiver or patient work productivity (Table 3). The largest improvement in work productivity was seen with conversion of trastuzumab IV to SC formulation for patients receiving maintenance therapy (34.7% increase). Work productivity for patients receiving trastuzumab in combination with chemotherapy or either type of rituximab therapy increased by 9.9 to 19.2%.

**Table 3:** Estimated caregiver productivity increase with subcutaneous versus intravenous monoclonal antibodies administration

	Under current conditions		Subcutaneous in all eligible patients^a^	
	Rituximab	Trastuzumab	Rituximab	Trastuzumab
In combination with chemotherapy	19.2%	9.9%	39.4%	22.5%
As maintenance therapy	17.0%	34.7%	22.9%	41.4%
Average^b^	21.2%		32.5%	

^a^Assuming a complete conversion to SC formulations according to the approved SmPC (45% of rituximab treatments and 91% of trastuzumab treatments)

^b^Based on the following assumptions: (1) the first dose of rituximab is administered IV in all patients; (2) 48% of the total number of patients have first-line follicular lymphoma and receive 8 cycles of rituximab in combination with chemotherapy and 12 as maintenance therapy, (3) 14% of the total number of patients have relapsed refractory follicular lymphoma and receive 8 cycles of rituximab in combination with chemotherapy and 8 as maintenance therapy, (4) 38% of the total number of patients have diffuse large B-cell lymphoma and receive 8 cycles of rituximab in combination with chemotherapy; (5) trastuzumab is given as first cycle loading dose in combination with chemotherapy, 7 subsequent cycles in combination with chemotherapy, and 10 cycles in maintenance therapy; a total of 18 cycles/year

*SC* subcutaneous, *SmPC* summary of product characteristics

#### Reduction in the use rate and time of venous access devices with reservoirs

In the case of trastuzumab, conversion to SC treatment also reduced the need for venous access devices with a reservoir (port-a-cath) in patients with early breast cancer (those with metastatic breast cancer have venous catheters inserted due to the need for multiple treatment lines, with the port-a-cath device being the preferred option at most of the assessed hospitals). For these early breast cancer patients, conversion from IV to SC trastuzumab administration allowed for the use of a peripherally inserted central catheter (PICC) instead of a port-a-cath device during the concomitant administration with chemotherapy at these hospitals, with removal of the PICC during maintenance therapy. Therefore, the rate of venous access device use during maintenance therapy was reduced by 81% and the time of use was reduced by 78%.

### Impact on capacity and quality of care of a hypothetical hospital that optimizes all identified processes with SC formulation

Table 4 shows the potential results that could be obtained from a hypothetical hospital if all of the identified improvements in efficiencies were implemented and the maximum possible conversion of IV rituximab (45%) and IV trastuzumab (91%) to SC formulations was adopted, according to their respective SmPC.

**Table 4:** Change in care capacity and quality assuming maximum SC administration efficiency and use

Parameter	Percent change in parameter
**Capacity of the hematology/oncology cancer treatment unit or day-stay hospital**	
1. Increase in treatments administered at the cancer treatment unit due to conversion from IV to SC	+ 5.75%
2. Increase in treatments administered due to rituximab premedication being administered in the waiting room	+ 0.40%
3. Increase in treatments that can be prepared and administered at the reference hospital because patients receiving SC can be treated at hospitals closer to their homes	+ 2.80%
4. Increase in treatments that can be prepared in the laminar flow hood at the hospital pharmacy:	
a. Due to conversion of IV to SC formulation	+ 12.16%
b. Due to preparation of SC formulations at the cancer treatment unit	+ 14.10%
**Quality**	
1. Reduction in time spent in hospital:	
a. For trastuzumab treatment	−60%
b. For rituximab treatment	−66%
2. Improvement in caregiver’s and/or patient’s work productivity	+ 32.5%

*IV* intravenous, *SC* subcutaneous

With maximum possible conversion of IV rituximab and trastuzumab to SC administration, the monthly capacity for treatment administration was estimated to increase by 5.75% as a result of the conversion from IV to SC formulations and the establishment of differential SC administration protocols. Administration of SC rituximab premedication in the waiting room was estimated to increase monthly administration capacity by 0.40%, and the transferal of SC trastuzumab administration to regional hospitals was estimated to increase monthly administration capacity by 1.50%.

With the maximum conversion of IV trastuzumab to SC administration, weekly capacity for preparation of additional treatments by the hospital pharmacy was estimated to increase by 12.16% as a result of the conversion of IV to SC administration, and by 14.10% as a result of transferring SC preparation to the cancer treatment unit. The transferal of SC trastuzumab administration to regional hospitals was estimated to increase the weekly capacity of the hospital pharmacy to prepare treatments under the laminar flow hood by 1.30%, according to trastuzumab SmPC indications.

According to these estimates, in a hypothetical hospital, the implementation of all processes to maximize the use of SC treatments in as many eligible patients as possible may increase the monthly capacity of the treating hospital by up to 23.1%. Mean hospital times per patient were estimated to decrease by 60% due to conversion from IV to SC trastuzumab (Fig. 2c) and by 66% due to conversion from IV to SC rituximab (Fig. 2d). This resulted in an estimated 32.5% increase in annual labor productivity for caregivers and active patients.

## Discussion

Previous studies have indicated that the use of SC versus IV formulations can reduce the time and costs involved in treatment [13]. However, to the best of our knowledge, the H-Excelencia study is the first to estimate the impact of conversion from IV to SC administration on hospital capacity and healthcare quality in the onco-hematology setting in Spain. This study showed that conversion from IV to SC administration of trastuzumab and rituximab improved onco-hematology hospital efficiency by increasing the capacity for treatment preparation and administration. Likewise, conversion to SC treatment reduced the time spent by patients at the hospital for treatment (with a positive impact on the patients’ and caregivers’ labor productivity), as well as reducing the utilization of IV access devices with a reservoir. The present study also showed that further improvements in hospital capacity and patient convenience were possible if the hospital implemented more efficient processes for drug preparation and administration (e.g., use of a dedicated chair for SC administration, administration of SC treatment at the nearest regional hospital whenever possible, and preparation of SC formulations outside of the laminar flood hood), together with maximizing the use of SC formulations of rituximab and trastuzumab whenever possible, according to their respective SmPC.

Previous studies have indicated that the use of SC rather than IV formulations of rituximab and trastuzumab is associated with reduced healthcare utilization and costs [12–17, 24–26]. In time and motion studies conducted alongside randomized clinical trials, rituximab SC and trastuzumab SC were associated with reductions in active HCP time (spent on both preparation and administration of rituximab SC and trastuzumab SC), as well as reduced time in the administration chair at the cancer treatment unit [13, 16]. With regard to reductions in healthcare utilization, the findings from a Spanish analysis of PrefHer study data were similar to those obtained in the current study: conversion from IV to SC trastuzumab administration led to a 50% reduction in active HCP time, and an 80% reduction in patient chair time [13].

Real-world studies have also consistently reported reductions in resource utilization and costs associated with conversion from IV to SC administration of rituximab and trastuzumab [12, 14, 15, 17, 24, 25]. While these studies had similar conclusions, the magnitude of the estimated savings differed between studies. Unlike our study, most of these aforementioned studies were based on the direct observation of both patients and HCPs rather than direct interviews with HCPs as in the current study, which might explain some of these differences. However, a recently published French observational study reported that conversion from IV to SC administration of trastuzumab and rituximab resulted in a 2.7% increase in the total number of chemotherapy sessions in the unit (with the potential to reach a 4.2% increase) [15], which is consistent with the 3.35% increase in the number of administered treatments seen in our study.

As described, most studies have used an observational methodology, but very few have been based on direct interviews with HCPs like the current study [24, 25]. Despite these studies being performed in different countries (Denmark [24] and Malaysia [25]), the HCP estimates of cost savings achieved with conversion from IV to SC trastuzumab formulation are quite consistent across these different sites.

The present study has several strengths. First, we used local data with direct input from HCPs involved in the treatment of patients (oncology, hematology, pharmacy, and nursing) to accurately reflect routine treatment practices, and the collected results are based on the aggregated data of the whole population of patients treated at the study sites. In addition, the identified efficiency indicators (Table 1) were developed after methodical and detailed fieldwork research consisting of face-to-face questionnaire-based interviews with HCPs from the same 10 hospitals where the study data were eventually collected. Finally, our analysis has estimated the maximum possible savings that can hypothetically be achieved by implementing processes to improve the efficiency of rituximab and trastuzumab administration and by using SC formulations as much as possible, based on the products’ approved indications. However, this study presents some limitations. First, the estimations of treatment and preparation time data by HCPs were obtained directly from the interviews, without being verified through direct observation, and could therefore be a source of recall bias. Nevertheless, since the estimated reduction in administration times observed in our study are consistent with those reported in similar studies in other countries [24, 25], we can conclude this limitation is unlikely to have significantly influenced our study results. Our study included 10 hospitals from evenly distributed geographic sites across Spain, and although they were of different sizes, and included both public and private institutions, our results would likely have varied if hospitals different to these had been included in the study. Thus our results may not be generalizable to all hospitals. While our study identified processes within participating hospitals that can be targeted to improve resource use, it is possible that other hospitals optimize different operational processes to those in our study. If these had also been included in our study, we might have observed an increased impact both in the capacity and quality of care compared with what was estimated in our study. Additional similar studies should be performed in the future to identify different hospital processes optimized across hospitals in Spain.

## Conclusions

This study has revealed new possibilities for improving resources and patient management in onco-hematology services. The use of SC rituximab and trastuzumab formulations improved the efficiency of the Spanish hospitals participating in the present study, increasing the monthly treatment administration and preparation capacity, decreasing administration times, and allowing for the optimization of processes. The quality of healthcare at these hospitals also improved by decreasing the average time of hospitalization and thus increasing the annual work productivity of patients and caregivers. Moreover, implementing similar processes and maximizing the use of SC formulations whenever possible could further enhance healthcare capacity, with an estimated 23.1% increase in the ability to administer other treatments and a > 60% reduction in time spent in hospital.

## Supplementary Information

**Additional file 1.** Discussion guides during fieldwork research. Additional file 1 describes how field research for this study was conducted. Specifically, it outlines the objectives of the study and the questions that each group of participants was asked.

## Data Availability

The datasets used and/or analysed during the current study, data associated and patient survey are available from the corresponding author on reasonable request.

## Abbreviations

HCP: Healthcare professional
IV: Intravenous
mAB: Monoclonal antibody
PICC: Peripherally inserted central catheter
SC: Subcutaneous
SmPC: Summary of product characteristics
