# Supplementary material for: Impact of the subcutaneous formulations of trastuzumab and rituximab on efficiency and resource optimization in Spanish hospitals: H-Excelencia study
Source: BMC Health Serv Res. 2021 Apr 8;21:320. doi: 10.1186/s12913-021-06277-8 (PMC8034176; doi:10.1186/s12913-021-06277-8)
Supplement: Supplementary file 1 — Additional file 1. Discussion guides during fieldwork research. Additional file 1 describes how field research for this study was conducted. Specifically, it outlines the objectives of the study and the questions that each group of participants was asked. [file 12913_2021_6277_MOESM1_ESM.docx]

# Discussion guides during fieldwork research

Prior to each interview, the interviewee was informed of the objectives of the project.

The project aims to demonstrate how the use of the subcutaneous formulation of MabThera and/or Herceptin improves the efficiency of hospitals, through:

o Optimisation of internal hospital processes to manage resources.

o Increasing the capacity of the onco-haematological day hospital to be able to treat more patients.

o Improvement of the quality of care in order to humanise onco-haematology services.

Below are the questions specific to each interviewed group.

## Nurses administering trastuzumab

In order to calculate and obtain information for the development of indicators to quantify the improvement in efficiency in hospitals, we will proceed to ask a series of questions about your centre:

1. What is the total volume of patients attended daily in your day hospital?
2. What are the opening hours of your day hospital?
3. How many management positions are there in your hospital's day hospital?
4. Number of armchairs with perfusion equipment
5. Number of armchairs without perfusion equipment (specific armchairs for subcutaneous administration)
6. Number of beds
7. Does your day hospital have a differentiated chair (without equipment associated with IV treatments) for the administration of subcutaneous treatments?
8. How many patients with Herceptin SC receive treatment in this differentiated chair? (only respond if a differentiated circuit for SC treatments has been implanted in your day hospital)
9. What is the average time of occupancy of administrative posts (armchair) for the following cases? - count the time from the time the patient is placed in the chair until he or she is lifted from the chair.
10. Intravenous treatment of Herceptin in combination with chemotherapy:

Loading dose.

Second infusions.

1. Intravenous treatment of Herceptin in maintenance therapy
2. Subcutaneous treatment of Herceptin in combination with chemotherapy
3. Subcutaneous treatment of Herceptin in maintenance therapy
4. What volume of patients are currently being treated at your day hospital with the following treatments?
5. Intravenous treatment of Herceptin in combination with chemotherapy:

Loading dose (new patients).

Maintenance dose.

1. Intravenous treatment of Herceptin in maintenance therapy
2. Subcutaneous treatment of Herceptin in combination with chemotherapy
3. Subcutaneous treatment of Herceptin in maintenance therapy
4. What type of pre-medication should be given with Herceptin?
5. If yes, what type of route of administration is used for the administration of pre-medication in the following treatments?
6. Intravenous treatment of Herceptin in combination with chemotherapy
7. Intravenous treatment of Herceptin in maintenance therapy
8. Subcutaneous treatment of Herceptin in combination with chemotherapy
9. Subcutaneous treatment of Herceptin in maintenance therapy
10. What is the average time of administration of Herceptin pre-medication in the following cases?
11. Intravenous treatment of Herceptin in combination with chemotherapy
12. Intravenous treatment of Herceptin in maintenance therapy
13. Subcutaneous treatment of Herceptin in combination with chemotherapy
14. Subcutaneous treatment of Herceptin in maintenance therapy
15. If the pre-medication of SC treatments is administered orally; where is the pre-medication administered to the patient; in the waiting room, in the nurse's office or in the infusion chairs?
16. Where is subcutaneous Herceptin prepared (in monotherapy and in combination with chemotherapy)?
17. In pharmacy in vertical laminar flow hoods
18. In pharmacy but outside the vertical laminar flow hoods
19. At the day hospital without a vertical laminar flow hood
20. Other
21. What is the average time of transfer of medication from the pharmacy to the day hospital by the warden?
22. What is the average preparation time by the nurse at the day hospital itself for the following treatments? (only to answer in the case that it is the case of your hospital)
23. Subcutaneous treatment of Herceptin in maintenance therapy
24. Subcutaneous treatment of Herceptin in combination with chemotherapy
25. Do you have a computer system that allows you to schedule treatments lasting less than 30 minutes?
26. Issues related to the IPCC or port-a-cath:
27. How many patients currently carry a PICC for the following treatments?
28. Herceptin IV:
29. Early breast cancer
30. Metastatic breast cancer
31. Herceptin SC:
32. Early breast cancer
33. Metastatic breast cancer
34. What volume of patients currently carry a port-a-cath for the following treatments?
35. Herceptin IV:
36. Early breast cancer
37. Metastatic breast cancer
38. Herceptin SC:
39. Early breast cancer
40. Metastatic breast cancer
41. For patients with PICC, when is the device removed in the following treatments? And why?
42. Herceptin IV:
43. Early breast cancer
44. Metastatic breast cancer
45. Herceptin SC:
46. Early breast cancer
47. Metastatic breast cancer
48. How long does it take to start treatment if I need PICC and if I need port-a-cath?
49. Under what criteria is it decided to use a reservoir in a treatment? Which professional makes this decision?
50. What is the unit cost associated with a port-a-cath / PICC (peripheral central insertion catheter)?
51. In relation to the port-a-cath / PICC (peripheral insertion central catheter); what materials and consumables are needed to place the device in a patient (including surgery if appropriate)? What is the unit cost of these?
52. What materials and consumables are necessary for the maintenance of a port-a-cath / PICC (peripheral insertion central catheter)? What is the unit cost of these?
53. What is the average time of placement of a port-a-cath / PICC (peripheral central insertion catheter) by the physician and nurse?
54. What percentage of patients in general are accompanied by a relative? What percentage of Herceptin patients are accompanied by a relative? Is there a difference when the treatment is subcutaneous compared to intravenous?
55. What is the average waiting time for the patient to go to the day hospital for the following treatments? - time from the time you identify yourself at the reception desk until you sit at the HDO booth or time from the time you leave the office until you sit at the HDO booth.
56. Intravenous treatment of Herceptin in combination with chemotherapy
57. Intravenous treatment of Herceptin in maintenance therapy
58. Subcutaneous treatment of Herceptin in combination with chemotherapy
59. Subcutaneous treatment of Herceptin in maintenance therapy
60. Where is blood drawn from patients?
61. In the laboratory of the reference hospital
62. Referral hospital day hospital
63. In the laboratory of the regional hospital closest to the patient's home
64. At the day hospital of the regional hospital closest to the patient's home
65. In the patient's primary care center
66. Other
67. What is the average time it takes for the patient to travel to the health center to carry out the analysis? By what means of transport do you usually do this? Who bears the cost of this transport (patient or hospital)?
68. What is the average time it takes for a patient to travel to his or her reference hospital? By what means of transport does this usually take place? Who bears the cost of this (patient or hospital)?
69. What is the average time it takes for the patient to travel to his/her local hospital? By what means of transport does he/she usually do so? Who bears the cost of this transport (patient or hospital)?
70. What is the average time from the extraction of the sample for analysis to the consultation? And from the consultation to the administration of treatment in the following cases?
71. Treatment where the analysis is carried out in the hospital
72. Treatment where the analysis is carried out in the Health Centre
73. How many patients with Herceptin SC treatment (in combination with chemotherapy and maintenance therapy) receive treatment at the county hospital closest to their residence?
74. Do you have a register of patient complaints and claims?

## Nurses administering rituximab

In order to calculate and obtain information for the development of indicators to quantify the improvement in efficiency in hospitals, we will proceed to ask a series of questions about your center:

1. What is the total daily patient volume at your day hospital?
2. What are the opening hours of your day hospital?
3. How many management positions are there in your hospital's day hospital?
4. Number of armchairs with perfusion equipment
5. Number of armchairs without perfusion equipment (specific armchairs for subcutaneous administration)
6. Number of beds
7. Does your day hospital have a differentiated chair (without equipment associated with IV treatments) for the administration of subcutaneous treatments?
8. How many patients with MabThera SC receive treatment in this differentiated chair (only respond if a differentiated circuit for SC treatments has been implanted in your day hospital)?
9. What is the average time of occupancy of the administrative posts (armchair) for the following cases? - count the time from the time the patient is placed in the chair until he or she is lifted from the chair.
10. Intravenous treatment of MabThera in combination with chemotherapy
11. Intravenous treatment of MabThera in maintenance therapy
12. Subcutaneous treatment of MabThera in combination with chemotherapy
13. Subcutaneous treatment of MabThera in maintenance therapy
14. What volume of patients are currently being treated at your day hospital with the following treatments?
15. Intravenous treatment of MabThera in combination with chemotherapy
16. Intravenous treatment of MabThera in maintenance therapy
17. Subcutaneous treatment of MabThera in combination with chemotherapy
18. Subcutaneous treatment of MabThera in maintenance therapy
19. What type of pre-medication should be administered with MabThera?
20. If yes, what type of route of administration is used for the administration of pre-medication in the following treatments?
21. Intravenous treatment of MabThera in combination with chemotherapy
22. Intravenous treatment of MabThera in maintenance therapy
23. Subcutaneous treatment of MabThera in combination with chemotherapy
24. Subcutaneous treatment of MabThera in maintenance therapy
25. What is the average time of administration of MabThera pre-medication in the following cases?
26. Intravenous treatment of MabThera in combination with chemotherapy
27. Intravenous treatment of MabThera in maintenance therapy
28. Subcutaneous treatment of MabThera in combination with chemotherapy
29. Subcutaneous treatment of MabThera in maintenance therapy
30. If the pre-medication of SC treatments is administered orally; where is the pre-medication administered to the patient; in the waiting room, in the nurse's office or in the infusion chairs?
31. Where is subcutaneous MabThera prepared (in monotherapy and in combination with chemotherapy)?
32. In pharmacy in vertical laminar flow hoods
33. In pharmacy but outside the vertical laminar flow hoods
34. At the day hospital without a vertical laminar flow hood
35. Other
36. What is the average time of transfer of medication from the pharmacy to the day hospital by the warden?
37. What is the average preparation time by the nurse in the day hospital itself for the following treatments? (only to answer in the case that it is the case of your hospital)
38. Subcutaneous treatment of MabThera in maintenance therapy
39. Subcutaneous treatment of MabThera in combination with chemotherapy
40. Intravenous treatment of MabThera in combination with chemotherapy
41. Intravenous treatment of MabThera in maintenance therapy
42. Do you have a computer system that allows you to schedule treatments lasting less than 30 minutes?
43. What percentage of patients in general are accompanied by a relative? What percentage of patients with MabThera are accompanied by a relative? Is there a difference when the treatment is subcutaneous compared to intravenous?
44. What is the average waiting time for the patient to go to the day hospital in the case of the following treatments? - time from the time you identify yourself at the reception desk to the time you sit at the HDO booth or time from the time you leave the office to the time you sit at the HDO booth.
45. Intravenous treatment of MabThera in combination with chemotherapy
46. Intravenous treatment of MabThera in maintenance therapy
47. Subcutaneous treatment of MabThera in combination with chemotherapy
48. Subcutaneous treatment of MabThera in maintenance therapy
49. Where is the blood sample taken from patients?
50. In the laboratory of the reference hospital
51. Referral hospital day hospital
52. In the laboratory of the regional hospital closest to the patient's home
53. At the day hospital of the regional hospital closest to the patient's home
54. In the patient's primary care centre
55. Other
56. What is the average time it takes for the patient to travel to the health center to carry out the analysis? By what means of transport does the patient usually do so? Who bears the cost of this transport (patient or hospital)?
57. What is the average time it takes for the patient to travel to the reference hospital? By what means of transport does the patient usually travel? Who bears the cost of this (patient or hospital)?
58. What is the average time it takes for the patient to travel to his or her local hospital? By what means of transport does he or she usually do so? Who bears the cost of this transport (patient or hospital)?
59. What is the average time from the extraction of the sample for analysis to the consultation? And from consultation to the administration of treatment in the following cases?
60. Treatment where the analysis is carried out in the hospital
61. Treatment where the analysis is carried out in the health center
62. How many patients with MabThera SC treatment (in combination with chemotherapy and maintenance therapy) receive the treatment at the county hospital nearest to their residence?
63. Do you have a register of patient complaints and claims?

## Oncologists prescribing trastuzumab

With the aim of calculating and obtaining information for the elaboration of indicators that allow quantifying the improvement of efficiency in hospitals, a series of questions will be asked about your center:

1. Could you describe to us at a high level the process from prescribing the treatment to completing the administration of the treatment?
2. Prescription of treatment.
3. Analytical.
4. Medical consultation.
5. Treatment preparation.
6. Administration of treatment.
7. What is the average time of a treatment initiation consultation with the physician (or nurse) for the following treatments? Are there differences if the treatment is subcutaneous or intravenous?
8. Intravenous treatment of Herceptin in combination with chemotherapy
9. Intravenous treatment of Herceptin in maintenance therapy
10. Subcutaneous treatment of Herceptin in combination with chemotherapy
11. Subcutaneous treatment of Herceptin in maintenance therapy
12. What is the average number of visits to the oncologist that the patient must go to during his/her treatment and what is the average duration of these consultations for the following treatments?
13. Intravenous treatment of Herceptin in combination with chemotherapy
14. Intravenous treatment of Herceptin in maintenance therapy
15. Subcutaneous treatment of Herceptin in combination with chemotherapy
16. Subcutaneous treatment of Herceptin in maintenance therapy
17. What is the average number of treatment cancellations per week due to unfavorable results from the analysis of the following treatments?
18. Intravenous treatment of Herceptin in combination with chemotherapy
19. Intravenous treatment of Herceptin in maintenance therapy
20. Subcutaneous treatment of Herceptin in combination with chemotherapy
21. Subcutaneous treatment of Herceptin in maintenance therapy
22. What percentage of patients are accompanied by a family member? (at global level and in the particular case of Herceptin)
23. What is the average waiting time for the patient from the time the test is performed to the time the patient comes for the following treatments?
24. Intravenous treatment of Herceptin in combination with chemotherapy
25. Intravenous treatment of Herceptin in maintenance therapy
26. Subcutaneous treatment of Herceptin in combination with chemotherapy
27. Subcutaneous treatment of Herceptin in maintenance therapy
28. What is the average time from sample extraction for analysis to medical consultation in the following cases?
29. Treatment where the test is performed in the hospital
30. Treatment where the analysis is carried out in the health center
31. What is the average waiting time on the part of the patient from the time he/she leaves the consultation until he/she goes to the day hospital in the case of the following treatments? - time from the time you identify yourself at the reception desk until you sit at the HDO booth or time from the time you leave the office until you sit at the HDO booth.
32. Intravenous treatment of Herceptin in combination with chemotherapy
33. Intravenous treatment of Herceptin in maintenance therapy
34. Subcutaneous treatment of Herceptin in combination with chemotherapy
35. Subcutaneous treatment of Herceptin in maintenance therapy
36. Where is the blood sample taken from patients and when?
37. In the laboratory of the reference hospital
38. Referral hospital day hospital
39. In the laboratory of the regional hospital closest to the patient's home
40. At the day hospital of the regional hospital closest to the patient's home
41. In the patient's primary care center
42. Other
43. What is the number of patients who have a reservoir (peripherally inserted central catheter)? - If yes, please indicate the type of reservoir
44. Intravenous treatment of Herceptin in combination with chemotherapy and maintenance therapy
45. Subcutaneous treatment of Herceptin in combination with chemotherapy and maintenance therapy
46. Under what criteria is it decided to use a reservoir in a treatment? Which professional makes this decision?
47. Occasionally, when the subcutaneous conversion is performed, the administration of the subcutaneous treatment in maintenance is diverted to regional hospitals close to the patients' homes, thus freeing up administration posts in the reference hospital; is this the case in your hospital? If so, answer the following questions:
    1. What is the average time it takes for the patient to travel to the health center to carry out the analysis? by what means of transport does the patient usually do so? Who bears the cost of this transport (patient or hospital)?
48. What is the average time it takes for the patient to travel to the reference hospital? By what means of transport does the patient usually travel? Who bears the cost of this transport (patient or hospital)?
49. What is the average time it takes for the patient to travel to his/her local hospital? By what means of transport does he/she usually do so? Who bears the cost of this transfer (patient or hospital)?
50. How many patients with Herceptin SC treatment (in combination with chemotherapy and maintenance therapy) receive the treatment at the county hospital closest to their residence?
51. Could you describe your overall view of the main advantages of the subcutaneous versus intravenous formulation?
52. Do you have a register of patient complaints and claims?

## Hematologists prescribing rituximab

In order to calculate and obtain information for the development of indicators to quantify the improvement in efficiency in hospitals, a series of questions will be asked about your center:

1. Could you describe to us at a high level the process from prescribing the treatment until the administration of the treatment is completed?
2. Prescription of treatment.
3. Analytical.
4. Medical consultation.
5. Treatment preparation.
6. Administration of treatment.
7. What type of pre-medication should be administered with MabThera?
8. If yes, what type of route of administration is used for the administration of pre-medication in the following treatments?
9. Intravenous treatment of MabThera in combination with chemotherapy
10. Intravenous treatment of MabThera in maintenance therapy
11. Subcutaneous treatment of MabThera in combination with chemotherapy
12. Subcutaneous treatment of MabThera in maintenance therapy
13. If the pre-medication of SC treatments is administered orally; where is the pre-medication administered to the patient; in the waiting room, in the nursing office or in the infusion chairs?
14. Do you have a computer system that allows you to schedule treatments lasting less than 30 minutes?
15. What is the average time of a treatment initiation consultation with the doctor (or nurse) for the following treatments? Are there differences if the treatment is subcutaneous or intravenous?
16. Intravenous treatment of MabThera in combination with chemotherapy
17. Intravenous treatment of MabThera in maintenance therapy
18. Subcutaneous treatment of MabThera in combination with chemotherapy
19. Subcutaneous treatment of MabThera in maintenance therapy
20. What is the average number of visits to the hematologist to which the patient must go throughout his/her treatment and what is the average duration of these consultations for the following treatments?
21. Intravenous treatment of MabThera in combination with chemotherapy
22. Intravenous treatment of MabThera in maintenance therapy
23. Subcutaneous treatment of MabThera in combination with chemotherapy
24. Subcutaneous treatment of MabThera in maintenance therapy
25. What is the average number of treatment cancellations per week due to unfavorable results from the analysis of the following treatments?
26. Intravenous treatment of MabThera in combination with chemotherapy
27. Intravenous treatment of MabThera in maintenance therapy
28. Subcutaneous treatment of MabThera in combination with chemotherapy
29. Subcutaneous treatment of MabThera in maintenance therapy
30. What percentage of patients are accompanied by a family member (globally and in the particular case of MabThera)?
31. What is the average waiting time for the patient from the time the test is performed to the time the patient comes for the following treatments?
32. Intravenous treatment of MabThera in combination with chemotherapy
33. Intravenous treatment of MabThera in maintenance therapy
34. Subcutaneous treatment of MabThera in combination with chemotherapy
35. Subcutaneous treatment of MabThera in maintenance therapy
36. What is the average time from sample extraction for analysis to medical consultation in the following cases?
37. Treatment where the test is performed in the hospital
38. Treatment where the analysis is carried out in the health center
39. What is the average waiting time on the part of the patient from the time he/she leaves the consultation until he/she goes to the day hospital in the case of the following treatments? - time from the time you identify yourself at the reception desk until you sit at the HDO booth or time from the time you leave the office until you sit at the HDO booth.
40. Intravenous treatment of MabThera in combination with chemotherapy
41. Intravenous treatment of MabThera in maintenance therapy
42. Subcutaneous treatment of MabThera in combination with chemotherapy
43. Subcutaneous treatment of MabThera in maintenance therapy
44. Where is the blood sample taken from patients?
45. In the laboratory of the reference hospital
46. Referral hospital day hospital
47. In the laboratory of the regional hospital closest to the patient's home
48. At the day hospital of the regional hospital closest to the patient's home
49. In the patient's primary care center
50. Other
51. Occasionally, when the subcutaneous conversion is performed, the administration of the subcutaneous treatment in maintenance is diverted to regional hospitals close to the patients' homes, thus freeing up administration posts in the reference hospital; is this the case in your hospital? If so, answer the following questions:
52. What is the average time it takes for the patient to travel to the health center to carry out the analysis? By what means of transport does the patient usually do so? Who bears the cost of this transport (patient or hospital)?
53. What is the average time it takes for the patient to travel to the reference hospital? By what means of transport does the patient usually travel? Who bears the cost of this transport (patient or hospital)?
54. What is the average time it takes for the patient to travel to his/her local hospital? By what means of transport does he/she usually do so? Who bears the cost of this transfer (patient or hospital)?
55. How many patients with Herceptin SC treatment (in combination with chemotherapy and maintenance therapy) receive the treatment at the county hospital closest to their residence?
56. Could you describe your overall view of the main advantages of the subcutaneous versus intravenous formulation?
57. Do you have a register of patient complaints and claims?

## For pharmacists

With the aim of calculating and obtaining information for the elaboration of indicators that allow quantifying the improvement of efficiency in hospitals, a series of questions will be asked about your center:

1. What volume of TOTAL daily patients are currently being treated in your day hospital?
2. What is the average volume of TOTAL daily preparations prepared at the hospital pharmacy for HDOH?
3. How many vertical flow bells are available in your hospital? What are their opening hours?
4. If your hospital is in a geographically dispersed region, who buys Herceptin and MabThera? The general hospital or the county hospital?
5. What volume of patients are currently being treated in your day hospital with the following treatments?
6. Intravenous treatment of MabThera in combination with chemotherapy
7. Intravenous treatment of MabThera in maintenance therapy
8. Intravenous treatment of Herceptin in combination with chemotherapy:
9. Loading dose (new patients).
10. Maintenance dose.
11. Intravenous treatment of Herceptin in maintenance therapy
12. Subcutaneous treatment of MabThera in combination with chemotherapy
13. Subcutaneous treatment of MabThera in maintenance therapy
14. Subcutaneous treatment of Herceptin in combination with chemotherapy
15. Subcutaneous treatment of Herceptin in maintenance therapy
16. What is the average volume of daily or weekly preparations of the following treatments?
17. Intravenous treatment of MabThera in combination with chemotherapy
18. Intravenous treatment of MabThera in maintenance therapy
19. Intravenous treatment of Herceptin in combination with chemotherapy
20. Intravenous treatment of Herceptin in maintenance therapy
21. Subcutaneous treatment of MabThera in combination with chemotherapy
22. Subcutaneous treatment of MabThera in maintenance therapy
23. Subcutaneous treatment of Herceptin in combination with chemotherapy
24. Subcutaneous treatment of Herceptin in maintenance therapy
25. MabThera and Herceptin subcutaneous (in monotherapy and in combination with chemotherapy)?
26. In pharmacy in vertical laminar flow hoods
27. In pharmacy but outside the vertical laminar flow hoods
28. At the Day Hospital without a vertical laminar flow hood
29. Other
30. What is the average preparation time in the vertical laminar flow hood for the following treatments? (If SC treatments are not prepared in a vertical laminar flow hood, do not comply with e, f, g, h).
31. Intravenous treatment of MabThera in combination with chemotherapy
32. Intravenous treatment of MabThera in maintenance therapy
33. Intravenous treatment of Herceptin in combination with chemotherapy
34. Intravenous treatment of Herceptin in maintenance therapy
35. Subcutaneous treatment of MabThera in combination with chemotherapy
36. Subcutaneous treatment of MabThera in maintenance therapy
37. Subcutaneous treatment of Herceptin in combination with chemotherapy
38. Subcutaneous treatment of Herceptin in maintenance therapy
39. What is the average preparation time outside the vertical laminar flow hood but in the hospital pharmacy service for the following treatments? (only answer if it is the case of your hospital)
40. Subcutaneous treatment of MabThera in maintenance therapy
41. Subcutaneous treatment of Herceptin in maintenance therapy
42. Subcutaneous treatment of MabThera in combination with chemotherapy
43. Subcutaneous treatment of Herceptin in combination with chemotherapy
44. What is the average preparation time at the day hospital for the following treatments? (answer only if it is the case of your hospital)
45. Subcutaneous treatment of MabThera in maintenance therapy
46. Subcutaneous treatment of Herceptin in maintenance therapy
47. Subcutaneous treatment of MabThera in combination with chemotherapy
48. Subcutaneous treatment of Herceptin in combination with chemotherapy
49. What is the average time of transfer of medication from the pharmacy to the day hospital by the warden?
50. What is the average number of treatment cancellations per week due to unfavorable results from the analysis of the following treatments?
51. Intravenous treatment of MabThera in combination with chemotherapy
52. Intravenous treatment of MabThera in maintenance therapy
53. Intravenous treatment of Herceptin in combination with chemotherapy
54. Intravenous treatment of Herceptin in maintenance therapy
55. Subcutaneous treatment of MabThera in combination with chemotherapy
56. Subcutaneous treatment of MabThera in maintenance therapy
57. Subcutaneous treatment of Herceptin in combination with chemotherapy
58. Subcutaneous treatment of Herceptin in maintenance therapy
59. What is the average number of preparation errors for the following treatments?
60. Intravenous treatment of MabThera in combination with chemotherapy
61. Intravenous treatment of MabThera in maintenance therapy
62. Intravenous treatment of Herceptin in combination with chemotherapy
63. Intravenous treatment of Herceptin in maintenance therapy
64. What is the average number of samples requiring re-preparation for the following treatments?
65. Intravenous treatment of MabThera in combination with chemotherapy
66. Intravenous treatment of MabThera in maintenance therapy
67. Intravenous treatment of Herceptin in combination with chemotherapy
68. Intravenous treatment of Herceptin in maintenance therapy
69. What is the average cost of one dose of the following treatments (please indicate the number of vials used and the unit cost associated with this where appropriate)?
70. Intravenous treatment of MabThera
71. Intravenous treatment of Herceptin
72. MabThera Subcutaneous Treatment
73. Subcutaneous treatment of Herceptin
74. What is the average time from the physician's confirmation of treatment to the administration of treatment for the following treatments?
75. Intravenous treatment of MabThera
76. Intravenous treatment of Herceptin
77. MabThera subcutaneous treatment
78. Subcutaneous treatment of Herceptin
